# Lymphome malin non hodgkinien primitif bilatéral du sein: à propos d'un cas

**DOI:** 10.11604/pamj.2015.20.234.6288

**Published:** 2015-03-12

**Authors:** Abderrahman El Mazghi, Kaoutar Loukili, Ayoub Mesnaoui, Issam Lalya, Touria Bouhafa, Hanan El Kacemi, Taieb Kebdani, Khalid Hassouni

**Affiliations:** 1 Faculté de Médecine et de Pharmacie, Université sidi Mohamed Ben Abdellah & Service de Radiothérapie, CHU Hassan II, Fès, Maroc; 2 Service de Radiothérapie, HIM Mohamed V, Rabat, Maroc; 3 Service de Radiothérapie, Institut National d'Oncologie, Rabat, Maroc

**Keywords:** Sein, lymphoma, non-hodgkinien, primitive, breast, lymphoma, Non-Hodgkin, primitive

## Abstract

Les lymphomes malins non-hodgkiniens (LMNH) primitifs du sein sont des tumeurs rares. Leur symptomatologie clinique est polymorphe. L'imagerie médicale est non-spécifique. Le diagnostic peut être évoqué à l'examen cytologique, sa confirmation est toujours histologique. Il s'agit essentiellement de lymphomes de type B, ceux de type NK/T restant rares. Les plus fréquents sont les lymphomes diffus à grandes cellules présentant la particularité de donner des rechutes sous forme d'extension au système nerveux central. Nous rapportons un cas de LMNH primitif bilatéral du sein chez une patiente âgée de 33 ans, révélé par deux nodules mammaires bilatéraux. La mammographie et l'examen extemporané ont évoqué une tumeur phyllode. Le diagnostic du LMNH n'a été fait qu'après examen histologique définitif. Sous chimiothérapie, l’évolution était favorable avec un recul de 15 mois.

## Introduction

L'atteinte primitive du sein par un lymphome malin non hodgkinien (LMNH) est une éventualité rare. Ces tumeurs ne représentent en effet que 0,04 à 0,5% des tumeurs malignes mammaires. Elles représentent entre 0,38% à 0,7% de tous les LMNH [1, 2]. A travers ce nouveau cas traité dans notre centre hospitalier et universitaire, nous allons discuter les aspects épidémiologiques, cliniques, histologiques et thérapeutiques de cette tumeur rare en se concentrant sur le rôle et l′efficacité de la chimiothérapie dans sa prise en charge.

## Patient et observation

**Figure 1:**
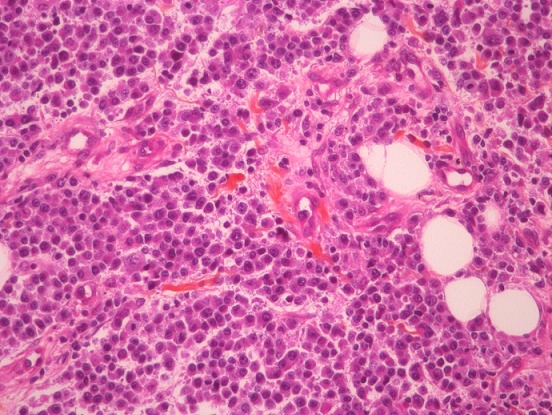
HES x 400: lobules de grandes cellules indifférenciées, d'aspect plasmocytoide à cytoplasme excentré évoquant un LMNH à grandes cellules B plasmoblastique

Il s'agit d'une patiente âgée de 33 ans, unigeste, unipare, qui s'est présentée en consultation dans un hôpital régional pour un nodule du sein gauche apparu depuis quatre mois, ayant augmenté progressivement de taille et un deuxième au niveau du sein droit découvert il ya deux semaines. L'examen clinique a trouvé une masse de 5 cm de grand axe au niveau du quadrant inféro-externe du sein gauche, de consistance élastique, mal limitée, indolore, non adhérente, et sans signes inflammatoires en regard. L'examen du sein droit a objectivé un nodule du quadrant supéro-externe de 3 cm de grand axe, ayant les mêmes caractéristiques sémiologiques que celui du sein controlatéral. Les aires ganglionnaires axillaires étaient libres. Le reste de l'examen somatique était normal. Par la suite la patiente a été vue lors d'une caravane médicale au cours de laquelle un examen extemporané de la masse du sein gauche est revenu en faveur d'un sarcome phyllode de haut grade. La patiente a bénéficié d'une mastectomie gauche et une biopsie-exérèse du nodule du sein droit. L'examen macroscopique des pièces de mastectomie et de tumorectomie a trouvé de multiples plages tumorales détruisant le tissu mammaire normal. L’étude histologique (Figure 1) a révélé que les nodules tumoraux droits et gauches sont tous identiques. Ils sont le siège d'une prolifération de grandes cellules indifférenciées, d'aspect plasmocytoide à cytoplasme excentré, parfois vacuolaire. Les noyaux sont de contour rond, avec chromatine dispersée. Ils sont également parsemés de cellules de plus grande taille, atypiques. Cette prolifération détruit ou déforme les canaux galactophoriques. Il s'agit d'une prolifération plasmoblastique. Le profil immuno-histochimique est celui d'un lymphome malin à grandes cellules B plasmoblastique: CD20-, CD3-, CD45+, CD138+ pour toutes les cellules, Kappa/Lambda négatifs, EBR+, Ki67 80%.

**Figure 2:**
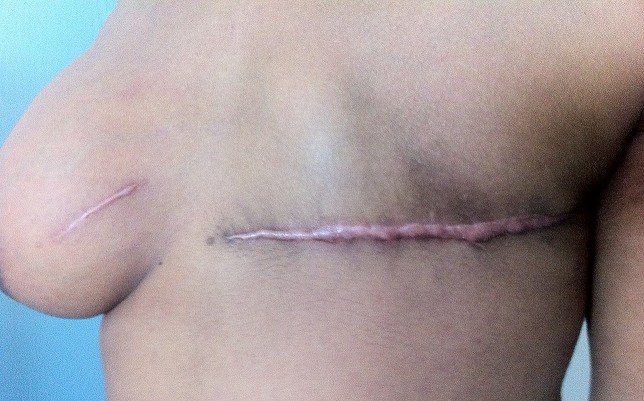
Patiente avec une cicatrice de mastectomie gauche et du tumorectomie droite (vue de profil)

**Figure 3:**
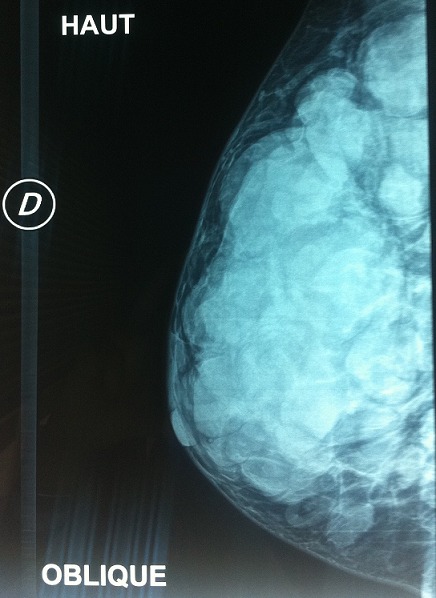
Mammographie montrant la présence au niveau du sein droit de multiples opacités de taille variable

**Figure 4:**
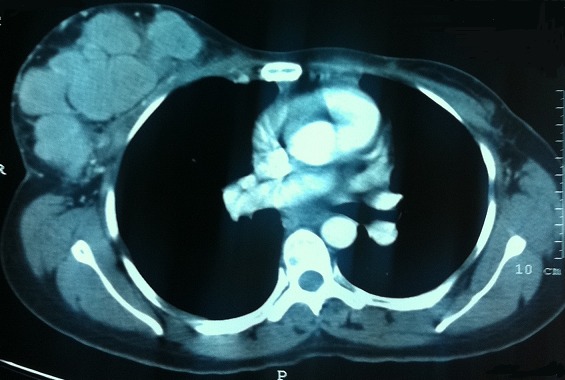
TDM thoracique montrant la présence au niveau du sein droit de multiples masses de taille variable (coupe axiale)

A l'admission au centre hospitalier universitaire Hassan II de Fès, on trouvait une patiente asymptomatique avec une cicatrice de la mastectomie gauche et de la tumorectomie droite (Figure 2). À la palpation, le sein droit est dur et polylobé. La mammographie a mis en évidence la présence au niveau du sein droit de multiples opacités de taille variable sans micro-calcifications mimant un aspect en lâcher de ballons (Figure 3). Le complément échographique a objectivé que ces opacités correspondaient à des formations tissulaires très hétérogènes, de contours irréguliers et de tailles variables. Un bilan d'extension à la recherche d'une localisation primitive de ce lymphome, est revenu négatif. La TDM cervico-thoraco-abdomino-pelvienne n'a montre que les masses mammaires droites déjà décrites sur la mammographie (Figure 4), la biopsie ostéo-médullaire était normale. La maladie a été classée IE selon la classification de Cotswold et la patiente a bénéficié de six cycles de chimiothérapie type CHOP (cyclophosphamide, Oncovin, Adriblastine, prédnisone) avec une bonne tolérance clinique et biologique. Elle est en rémission complète avec un recul de 15 mois.

## Discussion

On parle d'un lymphome primitif mammaire (LPM) lorsque le sein est le principal organe atteint, ou selon la majorité des cas, le seul site atteint par une prolifération lymphomateuse [3]. Wiseman a définit quatre critères pour poser le diagnostic d'un LPM [4]: prélèvement histologique adéquat; étroite association entre le tissu mammaire et l'infiltration lymphomateuse; absence de diagnostic de lymphome extra-mammaire; absence de métastases de la maladie à l'exception des adénopathies axillaires homolatérales.

En se basant, sur ces critères, le LPM est classé stade IE ou IIE (en cas d'association à une atteinte ganglionnaire axillaire homolatérale) selon la classification d'Ann Arbor modifié. Par conséquent, les patientes sont définies appartenant à un « stade précoce ». En revanche, la stadification des formes bilatérales est très controversée; certains auteurs, les ont classées stade IV, alors que d'autres les ont considérées stade IE ou IIE. Pour Hugh, il existe deux tableaux clinico-pathologiques: La forme unilatérale qui mime le carcinome du sein et intéresse la femme âgée alors que la forme bilatérale affecte plutôt les femmes jeunes, enceintes ou allaitantes, avec une dissémination rapide aux ovaires et au système nerveux central [5]. Notre patiente n'entre pas dans aucun des deux tableaux, puisqu'il s'agit d'une femme jeune, non enceinte et non allaitante, ayant présenté une forme bilatérale. Le signe d'appel clinique est une masse non douloureuse du sein dans 85% des cas [4–7]. Les symptômes systémiques B sont retrouvés dans 25 à 37% des cas. A la mammographie, il n'y pas de signe différenciant un lymphome d'un carcinome infiltrant.

Le diagnostic se fait par biopsie du sein. En reprenant les plus grandes séries publiées, le type histologique le plus fréquent est le lymphome diffus à grandes cellules B. Les lymphomes de bas grade du type MALT sont les deuxièmes types histologiques par ordre d'incidence [5]. Comme pour les LMNH ganglionnaires, une chimiothérapie incluant une anthracycline représente le pilier du traitement. Le schéma le plus administré est le CHOP. Cependant, l'effet de l'adjonction du rituximab (R) dans le traitement des LPM est incertain. Dans l’étude rétrospective de Zhao, comparant 31 patientes traitées par CHOP avec ou sans rituximab, le taux de survie à 5 ans était significativement meilleur dans le bras R-CHOP. En revanche, trois grandes études rétrospectives n'ont pas objectivé de bénéfice du rituximab; sauf que ces dernières se sont basées sur des anciens régimes de chimiothérapie [8]. La radiothérapie seule a été décrite [9] surtout dans les bas grades, les hauts grades ont été souvent traités par chimiothérapie ou une association radio-chimiothérapie. Les doses utilisées variaient de 30 à 45Gy. L'approche chirurgicale par mastectomie [6] a déjà été utilisée, mais n'a pas prouvé son efficacité et n'est pas indiquée [5].

La dissémination au système nerveux central varie de 5% à 29%, surtout pour les hauts grades. Certaines équipes proposent des injections intra-thécales prophylactiques de méthotrexate [7]. Quant à d'autres, ils préfèrent surveiller l'apparition de signes cliniques. Le pronostic des PLM ne diffère pas des LMNH ganglionnaires. Pour Giardini, le taux de survie est de 50% pour le stade IE et passe à la moitié (26%) pour les stades IIE. Par ailleurs, La survie retrouvée dans la littérature est très différente d'un auteur à l'autre faute d'inclusion de types histologiques comparables ainsi que la variabilité des traitements administrés [10].

## Conclusion

Les lymphomes malins primitifs du sein sont rares. Un bilan d'extension soigneux est nécessaire pour confirmer l'origine primitive. Le pronostic et le traitement rejoignent ceux des autres localisations lymphomateuses.
